# Relationships Between Neuromuscular Function and Functional Balance Performance in Firefighters

**DOI:** 10.1038/s41598-018-33555-z

**Published:** 2018-10-17

**Authors:** Jacob A. Mota, Timothy J. Barnette, Gena R. Gerstner, Hayden K. Giuliani, Andrew J. Tweedell, Craig R. Kleinberg, Brennan J. Thompson, Brian Pietrosimone, Eric D. Ryan

**Affiliations:** 1 Department of Exercise and Sport Science, University of North Carolina at Chapel Hill, Chapel Hill, NC USA; 2 Human Movement Science Curriculum, University of North Carolina at Chapel Hill, Chapel Hill, NC USA; 3 United States Army Research Laboratory, Aberdeen Proving Ground, MD USA; 4 Under Armour, Baltimore, MD USA; 5 Department of Kinesiology and Health Science, Utah State University, Logan, UT USA; 6 Department of Allied Health Sciences, University of North Carolina at Chapel Hill, Chapel Hill, NC USA

## Abstract

The purpose of the present study was to examine the relationships between neuromuscular function and functional balance performance in firefighters. Fifty career firefighters (35.1 ± 7.5 yr) performed isometric leg extension and flexion muscle actions to examine peak torque (PT), and absolute (aTQ) and normalized (nTQ; %PT) rapid torque variables at 50, 100, 150, and 200 ms. A performance index (PI) was determined from the functional balance assessment completion time. Partial correlations were used to examine the relationship between the PI and the maximal and rapid TQ variables for each muscle and the composite value, while controlling for demographic data related to the PI. Multiple regression analyses examined the relative contributions of the maximal and rapid aTQ variables, and demographic data on the PI. After controlling for age and %BF, the majority of the later aTQ and nTQ variables (100–200 ms) and PT were associated with the PI (*r* = −0.501–−0.315). Age, %BF, and aTQ_100_ explained 42–50% of the variance in the PI. Lower rapid strength, increased age, and poorer body composition were related to worse performance during the functional balance assessment. Strategies to improve rapid strength and %BF, especially in aging firefighters may impact dynamic balance abilities in firefighters.

## Introduction

Firefighters provide critical emergency services to communities across the country. However, due to the strenuous and dangerous nature of their jobs, they experience one of the highest rates of occupational injuries^1^, with an estimated annual cost between $2.8–$7.8 billion^2^. In 2016, over 62,000 injuries were reported in the United States with firefighters sustaining an injury approximately every 8 minutes, and nearly 40% of these injuries occurring during fireground operations^3^. One of the most common causes of fireground injuries in both structure^3^ and wildland^4^ firefighters are slips, trips, and falls (STFs). These injuries often result in higher than average worker compensation claims^5^ and increased worker absenteeism^6^, which is exacerbated in overweight and obese firefighters^7^.

Although the incidence and magnitude of STFs is well documented, very little is known regarding the specific risk factors contributing to STF-related injuries in firefighters. Kong, *et al*.^8^ has suggested that the cause of STFs includes extrinsic (e.g. personal protective equipment) or environmental factors (e.g. wet surfaces), as well as intrinsic or physiological factors. During fireground activities, the environment is often very unpredictable; however intrinsic or physiological factors are issues that are potentially modifiable. For example, in older adults maximal muscle strength has been suggested to be an important contributor in preventing falls^9^. However, the time needed to react and prevent a fall is shorter than the time required to reach maximal strength; therefore, the ability to generate force/torque rapidly may be critical in preventing STF-related injuries^10–12^. Furthermore, rapid torque production at early (i.e., 50 ms), but not late (i.e., 200 ms), time intervals have been suggested to be a better predictor of falls in the elderly^11^. However, these observations may also be muscle group specific as previous authors^10^ have demonstrated that the leg flexors are more predictive of previous falls history than the leg extensors.

Previous studies^13–17^ have developed an occupation specific assessment designed to determine a firefighter’s ability to maintain postural control while performing a simulated fireground task (i.e., walking on uneven surfaces, overhead obstacle avoidance) in an effort to examine firefighter specific STF risk factors. For example, Punakallio, *et al*.^18^ demonstrated that functional balance assessments were predictive of decreased work ability in firefighters over a 3-year period. These assessments have been altered from their original form to include tasks that may be more commonly encountered on the fireground^13^, and have been reported to be reliable^14^ and sensitive to changes in firefighter personal protective equipment (PPE) and self-contained breathing apparatus (SCBA)^13,19^. However, little is known about the relationship between neuromuscular function and STF-related injuries in the fire service. Determining how maximal and rapid strength (i.e., rapid torque) relates to functional balance performance may be important for identifying key impairments, which can be used to develop novel intervention strategies to mitigate the impact of STF in the fire service. Thus, the purpose of the present study was to examine the relationships between maximal and rapid strength and functional balance performance in career firefighters. Based upon previous literature^9–11,20^, we hypothesized that leg flexion rapid strength at early time intervals (i.e. 50 ms) would be the variable most strongly associated with functional balance performance.

## Methods

### Participants

**Figure 1:**
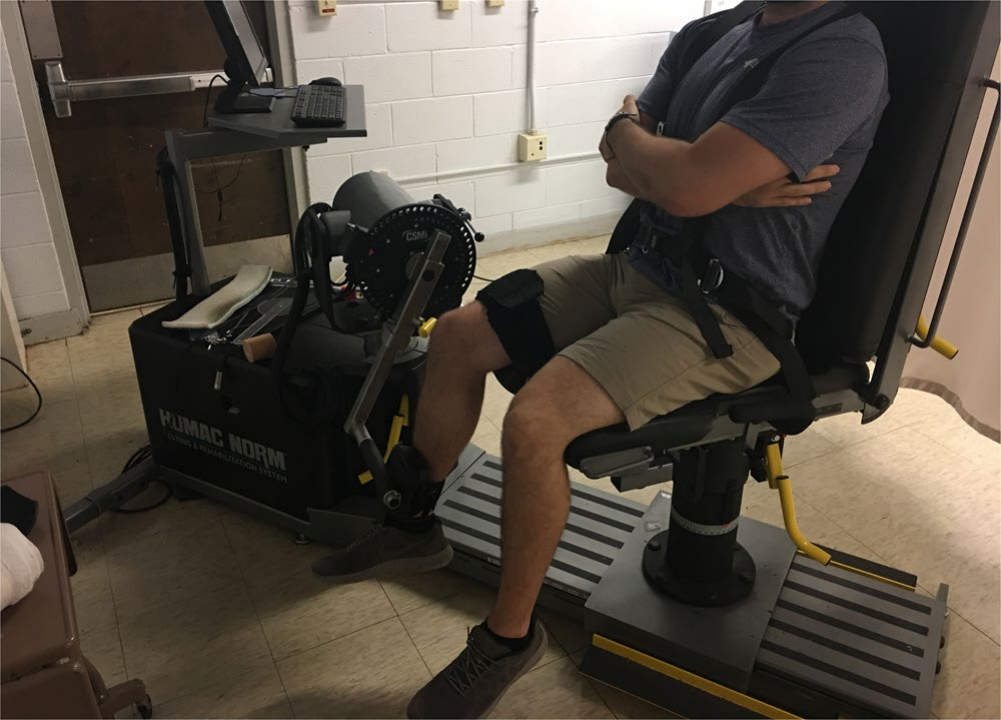
Example of isometric strength testing procedures used in the current study.

**Figure 2:**
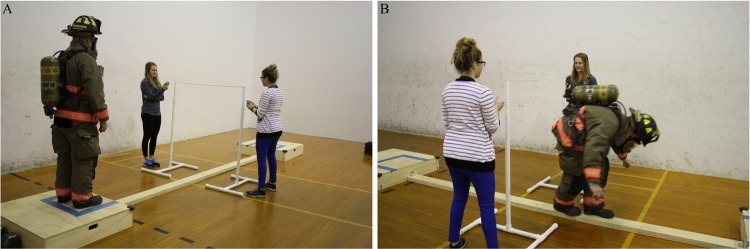
Example of the starting position (**A**) and overhead obstacle (**B**) in the functional balance assessment.

Fifty (43 males and 7 females) healthy career firefighters (mean ± SD age = 35.1 ± 7.5 years; stature = 178.8 ± 7.8 cm; mass = 94.5 ± 22.3 kg; body mass index [BMI] = 29.3 ± 5.5 kg/m^2^; percent body fat [%BF] =25.3 ± 5.4%) volunteered to take part in this study. Upon arrival to the laboratory and prior to participation, individuals read and signed an informed consent form for study participation and completed a health history questionnaire. Individuals were excluded from the study if they had a neuromuscular or metabolic disorder, or a current or recent (i.e., within three months) musculoskeletal injury to the lower back or leg. This study and its procedures were approved by the University of North Carolina at Chapel Hill institutional review board for the protection of human subjects. Informed consent was also provided for those included in the images for Figs 1 and 2. All tests were conducted according to the Declaration of Helsinki. The datasets generated and/or analyzed during the current study are available from the corresponding author on reasonable request.

### Research design

Participants visited the laboratory on three occasions, each separated by 2–10 days. The first visit served as a familiarization session during which the participants filled out the informed consent and health history questionnaire and practiced the isometric strength assessment. During the second visit, body composition and isometric strength were assessed, followed by the functional balance assessment familiarization. On the third visit, participants performed the functional balance assessment.

### Body composition

Participants arrived for their second visit in the morning following a minimum four hour fast. All participants were instructed to abstain from exercise in the 24 hours prior to testing. Percent body fat (%BF) was assessed using a calibrated dual energy X-ray absorptiometry (DXA, Hologic Discovery W, Bedford, MA) and all DXA scans were assessed using the included software (Hologic APEX Software, Version 3.3, Bedford, MA). Participants wore loose fitting clothing, were free from metal, and removed jewelry prior to scanning. Participants were then positioned supine and centered on the table in accordance with manufacturer instructions.

### Isometric strength assessment

The participants were seated on a calibrated commercial dynamometer (HUMAC Norm, Computer Sports Medicine Inc., Stoughton, MA) and were restrained with straps over the shoulders, hips, and thigh. The participant’s right knee joint was visually aligned with the input axis of the dynamometer and their right leg was extended to 60° below the horizontal plane (Fig. 1). The participants performed three submaximal (i.e., 50% of their perceived maximum) isometric contractions, to serve as a warm up, followed by three, 3–4 second maximal voluntary contractions (MVCs) of the leg extensors and flexors in random order, each separated by two minutes of rest. Participants were instructed to “push” or “pull” as fast and hard as possible without any countermovement or pretension, and strong verbal encouragement was provided. Each MVC was visually inspected for any countermovement or pretension as described by Gerstner *et al*.^21^. There was a noticeable countermovement or pretension for all attempted MVCs for five separate participants, one participant during leg extension and four participants during leg flexion. Consequently, these specific MVCs (one extension and four flexion) were discarded before all analyses. No participant had both a leg extension and flexion MVC that presented significant countermovement or pretension. The mean ± standard deviation (SD) slope of the baseline (200 ms prior to contraction onset) for the excluded leg extension and flexion MVCs were −9.23 Nm∙sec^−1^ and −10.64 ± 2.69 Nm∙sec^−1^, respectively. The baseline slopes for the remaining participants were −1.49 ± 2.06 Nm∙sec^−1^ for the leg extensors (n = 47) and −0.27 ± 1.40 Nm∙sec^−1^ for the leg flexors (n = 45). In addition, to determine baseline fluctuations, three standard deviations of the baseline mean were 0.29 ± 0.44 Nm which was 0.12 ± 0.16% MVC and 0.28 ± 0.34% MVC for the leg extensors and flexors, respectively. One participant’s leg extension MVCs was also removed as they were a significant outlier (>5 SDs from the mean). The MVC with the highest peak torque (PT) value was used for all subsequent analyses^22^.

### Signal processing

Torque (Nm) signals were sampled at 2.0 KHz with a Biopac MP150WSW data acquisition system and accompanying Acqknowledge software (Biopac System Inc., Goleta, CA) for each MVC. All signals were then stored on a personal computer (Lenovo IBM ThinkPad T420, Morrisville, NC) and processed offline with custom written software (LabVIEW 2014, National Instruments, Austin, TX). Torque signals were corrected for baseline passive tension and filtered with a zero-phase shift, fourth order low pass Butterworth filter (150 Hz)^23^. The identification of the torque onsets were determined per the recommendations of Maffiuletti *et al*.^24^ and similar to recent work^21^. The same experienced investigator (JAM) manually determined onset using a high-resolution x- and y-axis scale. The investigator zoomed in near signal onset and placed a vertical cursor at the point at which the respective signal deflected (i.e., last trough before signal deflection) from baseline^21,24^.

Peak torque (PT) was defined as the highest 500 ms epoch during the plateau of the MVC. The absolute and normalized (expressed as % of PT) rapid torque (TQ) variables were calculated from the torque-time curve at 50 ms (aTQ_50_; nTQ_50_), 100 ms (aTQ_100_; nTQ_100_), 150 ms (aTQ_150_; nTQ_150_), and 200 ms (aTQ_200_; nTQ_200_) from onset, similar to Gerstner, *et al*.^21^. These specific time points were chosen to represent early and late torque-time characteristics which have been suggested to represent unique physiological parameters^21,24,25^. Normalized TQ was also calculated as it is has been suggested to represent “qualitative” contractile characteristics independent of maximal strength^26^. Additionally, an average of the leg extension and flexion TQ values are reported as a composite measure of lower body maximal and rapid strength.

### Functional balance assessment

Functional balance was examined using a test similar to that recently developed by Hur *et al*.^13^. Previous authors have reported moderate-to-excellent test-retest reliability statistics (ICC_2,1_ range = 0.78–0.93) for a similar functional balance assessment^14^. The simulated firefighting task required firefighters to step down from a raised platform [1 m (L), 1 m (W), 20 cm (H)], walk along a narrow beam [4 m (L), 15 cm (W), 5 cm (H)], pass under an overhead obstacle, step up to a small defined space (45 × 45 cm^2^) on a second raised platform (identical in size to the original platform), and repeat the task walking backward as fast as possible and to stop within the same defined space on the original platform (Fig. 2). A lightweight wooden dowel (serving as the overhead obstacle) was placed at 75% of the participant’s height and designed to fall away if the rod was hit (Fig. 2). It is important to note that the current study utilized both a forward and backward walking task, versus just a forward walking task as described by Hur, *et al*.^13^. We choose to include backwards walking to 1) more closely mimic the potential demands during a live fire, and 2) backwards walking is more challenging and has been shown to be a more sensitive indicator of potential fall risks^27–29^. Participants were familiarized to the functional balance assessment on day two of testing. First, participants were instructed how to perform the assessment wearing only traditional work-out attire (i.e., loose fitting shirt, shorts, and athletic shoes). Once the participants were comfortable with the task, investigators instructed the participants to perform the assessment while wearing a SCBA (Mine Safety Appliance, 2216 psi, 30 minute cylinder), excluding the face piece. On the final testing day, participants initially practiced the assessment once wearing their own bunker gear. All subsequent time trials were performed with each participants full PPE which included their helmet, full bunker gear (minus gloves), and a SCBA (face piece and air cylinder). The SCBA was provided and identical for all participants. The participants completed five timed trials with a one minute rest period between each trial. For each trial, participants were instructed to complete the task as quickly as possible without falling off the plank or touching the floor (i.e., committing an error, as described below) and to self-select their method of avoiding the overhead obstacle. Three investigators were present during the balance assessment. Two investigators were responsible for timing each trial using a stopwatch, and monitored the participant during the testing to ensure participant safety and to replace the wooden dowel if it fell during the initial portion of the test (i.e. when performed walking forward). A third investigator was responsible for the verbal commands to begin and end each trial, in addition to capturing digital video (Samsung HMX-F90, Seoul, South Korea) of the entire assessment which was used to determine the number of minor and major errors following testing (described below). The command “three, two, one, GO” was given and the time started on ‘GO’. Time was stopped when the subject placed both feet back in the original starting position (within the defined marked area) on the first platform. The average of the times recorded by each of the two researchers was used as the performance time for each individual trial.

The number of major and minor errors and time to complete the assessment were used to determine a performance index (PI; lower PI indicative of better performance) as described by Hur, *et al*.^13^. All digitally recorded assessments were analyzed following testing by the same investigator (TJB) to determine the number of major and minor errors. A minor error was counted when: 1) a foot or hand contacted the ground, 2) a hand contacted a platform, 3) the subject stepped outside of the defined marked area on the platforms, or 4) the obstacle was touched but did not fall. A major error was counted when: 1) the obstacle was contacted and the rod fell, 2) both feet contacted the ground, or 3) the subject tripped or fell during the trial. A minor error counted as a one second penalty and a major error equaled a two second penalty which was added to the total time of each trial. Each subject’s best PI score was used for all analyses^14^. The PI was determined by the following equation described by Hur, *et al*.^13^:

$$PI=[time\,to\,complete\,trial\,(seconds)]+[number\,of\,minor\,errors\times 1]+[number\,of\,major\,errors\times 2]$$

### Statistical analysis

Pearson product-moment correlation coefficients (*r*) were calculated to examine the relationship between the PI and demographic data that included age, BMI, %BF, the weight of total equipment (PPE and SCBA), and the weight of total equipment expressed as a percentage of body mass. Subsequent partial correlations were used to examine the relationship between the PI and all maximal and rapid TQ variables for the leg extensors, flexors, and composite data, while controlling for demographic data related to the PI (i.e. age and %BF). Similar partial correlations were used to examine the relationship between all rapid TQ time points. Finally, three separate stepwise multiple regression analyses were utilized to determine the relative contributions of age, %BF, and maximal (PT) and rapid absolute strength variables (aTQ_50,_ aTQ_100,_ aTQ_150,_ aTQ_200_) on the PI for the extensors, flexors, and composite scores, respectively. It is important to highlight that the regression models were not forced to stop after any pre-determined number of predictor variable inputs. Multicollinearity was monitored using the variance inflation factor (VIF). An alpha level of *P* ≤ 0.05 was used to determine statistical significance for both analyses. All descriptive statistics are presented as mean ± SD. Also, partial correlation coefficients are presented as a range, where appropriate. All statistical procedures were performed using SPSS software (version 19.0, IBM SPSS Inc., Chicago, IL).

## Results

All torque variables are displayed in Table 1. There was a significant relationship between the PI and age (*r* = 0.406; *P* = 0.004) and %BF (*r* = 0.401; *P* = 0.004), but not with BMI, weight of total equipment, or weight of total equipment expressed as a percentage of body mass (*r* = −0.05–0.242; *P* ≥ 0.093).

**Table 1:** The mean ± SD and range of values for absolute and normalized torque.

Extension	Mean ± SD	Range	Flexion	Mean ± SD	Range	Composite	Mean ± SD	Range
**Absolute**			**Absolute**			**Absolute**		
aTQ_50_ (Nm)	10.38 ± 12.28	0.65–67.09	aTQ_50_ (Nm)	7.15 ± 6.66	0.42–25.57	aTQ_50_ (Nm)	8.79 ± 8.40	0.72–40.52
aTQ_100_ (Nm)	79.00 ± 47.32	3.82–192.46	aTQ_100_ (Nm)	33.77 ± 24.77	0.90–97.67	aTQ_100_ (Nm)	54.87 ± 32.70	2.36–139.76
aTQ_150_ (Nm)	116.88 ± 49.41	18.97–230.98	aTQ_150_ (Nm)	61.90 ± 33.80	1.89–146.62	aTQ_150_ (Nm)	88.01 ± 38.40	10.43–169.48
aTQ_200_ (Nm)	140.55 ± 7.56	18.97–230.98	aTQ_200_ (Nm)	67.41 ± 18.47	9.43–104.00	aTQ_200_ (Nm)	108.67 ± 39.24	15.32–207.75
PT (Nm)	234.45 ± 59.95	90.77–361.78	PT (Nm)	118.64 ± 31.28	50.25–172.17	PT (Nm)	174.07 ± 42.39	82.99–253.23
**Normalized**			**Normalized**			**Normalized**		
nTQ_50_ (%)	4.27 ± 4.39	0.24–24.07	nTQ_50_ (%)	5.71 ± 4.71	0.30–19.39	nTQ_50_ (%)	5.03 ± 3.89	0.70–17.19
nTQ_100_ (%)	32.99 ± 18.09	2.30–74.47	nTQ_100_ (%)	27.64 ± 18.29	1.19–74.89	nTQ_100_ (%)	29.88 ± 15.35	2.70–74.68
nTQ_150_ (%)	49.40 ± 16.99	10.47–94.49	nTQ_150_ (%)	50.51 ± 21.65	2.49–90.85	nTQ_150_ (%)	49.87 ± 16.67	11.70–85.93
nTQ_200_ (%)	59.44 ± 16.34	19.22–88.71	nTQ_200_ (%)	67.41 ± 18.47	9.43–104.00	nTQ_200_ (%)	62.91 ± 14.73	17.65–91.36

PT - peak torque; aTQ - absolute torque; nTQ - normalized torque.

nTQ data are expresed as a % of PT.

### Relationship between the performance index and isometric torque variables

For leg extension (Table 2), PI was related to aTQ_100,_ aTQ_150_, aTQ_200,_ and PT (*r* = −0.462–−0.362; *P* ≤ 0.05), but not aTQ_50_ (*r* = −0.068; *P* = 0.657). Similarly, PI was related to nTQ_100_, nTQ_150_, and nTQ_200_ (*r* = −0.405–−0.336, *P* ≤ 0.05), but not nTQ_50_ (*r* = −0.036; *P* = 0.813).

**Table 2:** Partial correlation coefficients (*r*) between the performance index and torque time intervals for leg extension, flexion, and composite values, when controlling for age and % body fat.

	Extension	Flexion	Composite
**Absolute**			
aTQ_50_	−0.068	−0.258	−0.117
aTQ_100_	−0.460^‡^	−0.384^*^	−0.482^‡^
aTQ_150_	−0.462^‡^	−0.265	−0.414^*^
aTQ_200_	−0.438^*^	−0.284	−0.413^*^
PT	−0.362^*^	−0.168	−0.279
**Normalized**			
nTQ_50_	−0.036	−0.288	−0.223
nTQ_100_	−0.405^*^	−0.410^*^	−0.501^‡^
nTQ_150_	−0.359^*^	−0.279	−0.389^*^
nTQ_200_	−0.336^*^	−0.315^*^	−0.414^*^

^*^Indicates *P* value ≤ 0.05; ^‡^Indicates *P* value ≤ 0.001.

PT - peak torque; aTQ - absolute torque.

nTQ - normalized torque (% of PT).

For leg flexion (Table 2), PI was related to aTQ_100_ (*r* −0.384; *P* = 0.011), but not aTQ_50_, aTQ_150_, aTQ_200,_ or PT (*r* = −0.284–−0.168; *P* ≥ 0.05). Furthermore, PI was related to nTQ_100_ (*r* = −0.410; *P* = 0.006) and nTQ_200_ (*r* = −0.315; *P* = 0.040) but not nTQ_50_ (*r* = −0.288; *P* = 0.061) or nTQ_150_ (*r* = −0.279; *P* = 0.070).

For the composite values (Table 2), PI was related to aTQ_100_, aTQ_150_, and aTQ_200_ (*r* = −0.482–−0.413; *P* ≤ 0.05) but not aTQ_50_ (*r* = −0.177; *P* = 0.268) or PT (*r* = −0.279; *P* = 0.078). Similarly, significant relationships were found between the PI and nTQ_100_, nTQ_150_, nTQ_200_ (*r* = −0.501–−0.389; *P* ≤ 0.05), but not nTQ_50_ (*r* = −0.223; *P* = 0.162).

### Predictability of the performance index

The results from the three separate stepwise multiple regression analyses (extensors, flexors, and composite) indicated that age, %BF, and aTQ_100_ were the best predictors of the PI (R^2^ = 0.421–0.502; *P* ≤ 0.05; maximum VIF = 1.039). The predictive regression equations are presented in Table 3.

**Table 3:** Unstandardized (Β, Β standard error [SE]) and standardized (*β*) regression coefficients for predicting PI from age, %BF, and aTQ_100_.

	Β	Β SE	*β*	t	*P*-Value	VIF	R^2^ Change	R^2^
***Model 1 – Extension***								
Constant	3.395	2.116	—	1.605	0.116	—		
Age	0.138	0.044	0.359	3.129	0.003	1.039	0.208	
aTQ_100_	−0.024	0.007	−0.387	−3.399	0.001	1.026	0.120	
%BF	0.192	0.060	0.364	3.185	0.003	1.031	0.128	0.456
***Model 2 – Flexion***								
Constant	1.948	2.272	—	0.857	0.396	—		
Age	0.166	0.047	0.419	3.507	0.001	1.013	0.204	
%BF	0.195	0.065	0.358	2.990	0.005	1.014	0.118	
aTQ_100_	−0.039	0.014	−0.317	−2.661	0.011	1.002	0.100	0.421
***Model 3 – Composite***								
Constant	2.821	2.075	—	1.360	0.182	—		
Age	0.160	0.043	0.428	3.734	0.001	1.027	0.257	
aTQ_100_	−0.035	0.010	−0.394	−3.435	0.001	1.029	0.115	
%BF	0.191	0.060	0.367	3.191	0.003	1.038	0.130	0.502

PI – performance index; %BF - % body fat; aTQ_100_ – absolute torque at 100 ms.

VIF – variance inflation factor.

## Discussion

Slips, trips, and falls remain one of the primary causes of injury in the fire service^3^, however little is known regarding the specific risk factors contributing to STF-related injuries. The primary findings of the present study indicated that: (1) age and %BF are positively correlated with the PI; (2) when controlling for age and %BF, rapid TQ production between 100–200 ms were negatively correlated to the PI, however, these correlations were muscle group specific; and (3) age, %BF, and aTQ100 had a cumulative effect on the prediction of the PI in career firefighters.

### Demographic data related to the PI

Previous studies have examined the relationships between age and similar functional balance assessments in firefighters^8,15,17^. For example, Punakallio^15^ demonstrated that functional balance performance decreased with increasing age. In a subsequent study, the same laboratory^17^ noted that the older firefighters (43–56 yrs) had poorer PI scores when compared to their younger (33–38 yrs) colleagues, however, these were not statistically significant (*P* = 0.065). These findings are in agreement with the results of the current study that demonstrated higher PI scores (poorer performance) were associated with increases in age. However, these are in contrast to a more recent study^8^ that reported no significant relationship between age and the PI. A potential cause for the differences between this study and our data may be the larger age range of the participants in our study (19–43 yrs vs. 20–50 yrs).

Previous studies have examined the relationships between increased adiposity and the risk of STFs and have suggested that increases in BMI may be related to an increased risk of falls and fall-related injuries^30–34^. Using a similar balance assessment to the current study, Kong, *et al*.^8^ reported no significant relationship between BMI and balance. These findings are similar to the present study, however, poorer PI scores were associated with increased %BF values (*r* = 0.401). Although we are aware of no additional studies that have examined the relationship between %BF and functional balance performance, previous authors have indicated that poor performance on firefighter specific tasks are related to increased %BF^35,36^. Thus, it is possible that %BF may more accurately reflect the obesity status of firefighters and the subsequent biomechanical and physiological consequences^32^ associated with obesity that may influence functional balance performance. This was suggested recently by Jitnarin, *et al*.^37^ who reported that the prevalence of obesity in firefighters is higher using %BF estimates than BMI. Furthermore, the addition of the PPE and SCBA have been shown to influence functional balance^8^, thus is it possible that the total mass of the equipment and relative (total equipment mass expressed as a percentage of body mass) external load may impact the PI scores. However, the results from the present study did not reveal any significant relationships between the PI and the total mass of the equipment or the relative external load.

### Maximal and rapid torque variables related to the PI

Previous studies have demonstrated that maximal and rapid strength are important predictors of fall history^9–11,20^. However, recent studies^10,11^ have suggested that rapid strength may be a better discriminator of balance recovery during a fall. For example, Bento, *et al*.^10^ demonstrated that older women with a history of falls had lower rate of torque development (RTD) but similar PT values than those without a history of falls, and that lower RTD values were associated with a greater number of falls. Palmer, *et al*.^11^ added to these findings indicating that elderly women without a falls history had significantly greater absolute and normalized early RTD (0–50 ms) but similar absolute and normalized late RTD (100–200 ms) and PT when compared to women with a history of falls. Based on these findings, we hypothesized that rapid strength, specifically during early time intervals (50 ms), may be more important and more strongly associated to functional balance performance than PT and the later rapid TQ variables. The primary findings of the current study demonstrated that the majority of the later absolute and normalized rapid TQ variables (100–200 ms) and PT (only during leg extension) were associated with the PI, whereas none of the early (50 ms) absolute and normalized rapid TQ variables were related to the PI. Furthermore, the stepwise regression analyses indicated that TQ at 100 ms (for both muscles) was the most significant predictor of the PI among all the strength variables, which was further supported by the composite values representing an overall lower body strength variable (Table 3). These findings support our hypothesis that rapid strength is associated with the PI and may be potentially more important than PT. However, our findings do not support the notion that the early rapid TQ variables (i.e. 50 ms) are the strongest predictors of functional balance performance. It is possible our current results may not specifically support the findings of Palmer, *et al*.^11^ due to the contrasting onset detection methods used. As discussed in a recent study^21^, automated methods to determine contraction onset (as done in the Palmer, *et al*.^11^ study) will typically occur later than the systematic manual onset procedure (used in the current study) suggested in the recent review by Maffiuletti, *et al*.^24^. The ability to discern between fallers and non-fallers with rapid strength characteristics of the lower body may also be muscle group specific. For example, Bento, *et al*.^10^ reported that RTD in the leg flexors, but not the leg extensors, was greater in the older women who had no history of falls. As such, we hypothesized that rapid TQ variables in the leg flexors, but not extensors, would be significantly correlated to the PI. Interestingly, our results indicated that all the leg extension absolute and normalized later rapid TQ variables (100–200 ms) were related to the PI, whereas fewer later rapid TQ variables of the leg flexors were related to the PI and these relationships were generally weaker than those reported for leg extension. While our findings may not fully support our original hypothesis, it is important to highlight the inclusion of the backwards walking task in our functional balance assessment. Backwards walking has previously been shown to have dissimilar patterns of muscle activation when compared to forwards walking^38,39^. Specifically, Thorstensson^38^ suggested that backwards walking relies upon the leg extensors more than during forwards walking. During the present investigation, the requirement to walk backwards may have significantly increased the difficulty of the functional balance assessment, increasing the importance of leg extensor rapid strength on the PI.

### Relative relationships between age, %BF, and rapid strength on the PI

The results of our stepwise regression analyses indicated that age, %BF, and aTQ100 predicted 42–50% of the variance in the PI. These findings are significant given approximately 50% of the fire service is over 40 years old^40^ and the prevalence of overweight and obesity exceeds that of the general public^7^. Fortunately, %BF and rapid strength are modifiable risk factors that can be improved with well-designed exercise and nutritional interventions. Future, more comprehensive studies are needed to determine the remaining unexplained variance (~50%) that may influence the PI. Specifically, previous studies have suggested that vision^41^, lower extremity coordination^42^, and/or physical activity habits^12^ may significantly impact the amount of explained variance in our functional balance assessment. Furthermore, additional investigations may wish to utilize a similar functional balance assessment while including laboratory induced slipping^16,32^ to further understand the mechanisms of STF recovery and/or the utility of this assessment to predict STF-related injuries. Lastly, although the current dynamometry setup is common when examining maximal and rapid strength characteristics, future studies may wish to examine strength at a posture where most STFs occur, similar to those described previously when examining injury specific mechanisms^43^.

In summary, the primary findings of the current investigation indicated that increased age and %BF were related with poorer performance on the functional balance assessment. When controlling for these variables, rapid strength (i.e., late rapid TQ production) displayed a negative relationship with our measure of balance, although this was found to be muscle group specific. The results from the stepwise multiple regression analyses suggest that age, %BF, and late rapid TQ production explain a significant (42–50%) amount of the variance in functional balance performance in career firefighters. These findings may be impactful to fire service administrators highlighting the need to integrate specific strategies to improve %BF and rapid strength, especially in aging firefighters.
